# Development and Assessment of Innovative High-Fidelity Simulation Vaccination Course Integrating Emergency Cases for Pharmacy Undergraduates—A Randomized Controlled Study

**DOI:** 10.3390/vaccines11020324

**Published:** 2023-01-31

**Authors:** Shahzad Ahmad Sayyed, Ahmed Reda Sharkas, Bushra Ali Sherazi, Armin Dabidian, Holger Schwender, Stephanie Laeer

**Affiliations:** 1 Institute of Clinical Pharmacy and Pharmacotherapy, Heinrich Heine University Duesseldorf, Universitaetsstrasse 1, 40225 Duesseldorf, Germany; 2 Institute of Pharmacy, Faculty of Pharmaceutical and Allied Health Sciences, Lahore College for Women University, Lahore 54000, Pakistan; 3 Mathematical Institute, Heinrich Heine University Duesseldorf, Universitaetsstrasse 1, 40225 Duesseldorf, Germany

**Keywords:** vaccination, pharmacy education, high-fidelity simulation, vaccination training

## Abstract

Recently, pharmacists in Germany were allowed to administer influenza and COVID-19 vaccines for people aged 12 years and older in order to increase vaccination coverage rates. In order to adapt the pharmacy curriculum for clinical practice, an innovative, vaccination training course using a high-fidelity simulator (HFS) was developed, implementing clinical scenarios to manage adverse events. In a randomized controlled trial using a pre and post design with pharmacy undergraduates, the intervention group interacted with an HFS, while the control group was trained with low-fidelity injection pads. Before and after the respective training, each participant went through an objective structured clinical examination (OSCE) and completed a self-assessment questionnaire and knowledge quiz. Both training methods showed a significant increase in skills, but there was also a significant greater increase in the intervention group when compared to the control group, particularly with respect to the vaccination process. Furthermore, every individual in the intervention group improved from the pre- to post-training OSCEs. Therefore, HFS has been proven to be an appropriate tool to train pharmacy students for the purposes of vaccine administration and to prepare for future challenges. Particularly, recognizing and managing adverse reactions can be addressed in a very effective way.

## 1. Introduction

Vaccination is the best and most successful method with respect to preventing infectious diseases [1]. In addition to reducing significant morbidities, disabilities, and mortalities, extensive immunization also possesses non-health benefits, such as improved cost-effectiveness, less of a disease burden, and increased educational achievements among children—due to their subsequent improved health [2,3,4,5]. There has been a substantial need for mass vaccinations since the COVID-19 pandemic began [6,7]. Similar to actions taken against other infectious diseases, immunizing a large number of people through numerous vaccinations is the main purpose. Furthermore, the immunization process is conducted for multiple reasons, including protecting those who cannot receive immunizations [8,9]. A vaccine coverage rate of 60–70% for COVID-19 was initially estimated, although this estimate was subject to change once cases of waning immunity as well as reinfections were reported [9,10]. Therefore, in response to this issue, it was recommended to perform repetitive booster shots [10,11]. As of October 2022, 64.8 million people (77.8% of the population) have received at least one vaccination dose in Germany. Of these, 63.5 million people (76.3%) have already received basic immunization. A total of 51.8 million people (62.2%) have also received a booster vaccination. Moreover, a sum of 8.3 million people (10.0%) have already received a second booster vaccination [12]. Currently, further vaccination is recommended for special population groups, such as people over 60, as well as immunocompromised persons or those working in healthcare institutions [11]. 

In order to address this demand for immunizations, physicians, veterinarians, dentists, and pharmacists were all asked to support the vaccination program in Germany [13]. However, in contrast to other countries—such as Argentina, the US, the UK, and Portugal—German pharmacists were not authorized to administer vaccines until 2020 [14,15]. It was only until recently, in 2020, that special pharmacist groups were authorized to vaccinate for flu in Germany [16,17]. This was allowed due to the successful vaccination programs that were implemented in about 26 countries around the world [15]. The legal stipulations for immunization administration in pharmacies were established in Argentina as early as 1983. Moreover, in 1996, pharmacists were authorized to administer the influenza vaccine in 14 American states. Pharmacy-based immunization was first introduced in Europe by the UK in 2002, followed by Portugal and Ireland [14]. The influenza vaccination is the most common vaccine administered by pharmacists worldwide [15]. The reason for this is due to the fact that influenza causes local outbreaks and seasonal epidemics all over the world. These outbreaks and epidemics occur due to the constant changes in the viral genome. As such, new vaccines are required and are approved every year, due to demand with respect to an annual booster vaccination [18]. Most pharmacists support the WHO’s target of a 75% vaccination rate for people aged 65 and over or for other risk groups [19,20]. This is due to the fact that retail pharmacies are considered to be the most accessible health care facilities [21]. It must be noted that the involvement of pharmacists in the vaccination process has resulted in an increased coverage rate, awareness, and education [22,23,24,25]. This was most particularly the case for those groups of people who cannot, or do not, want to be reached by conventional means [26]. 

**Figure 1:**
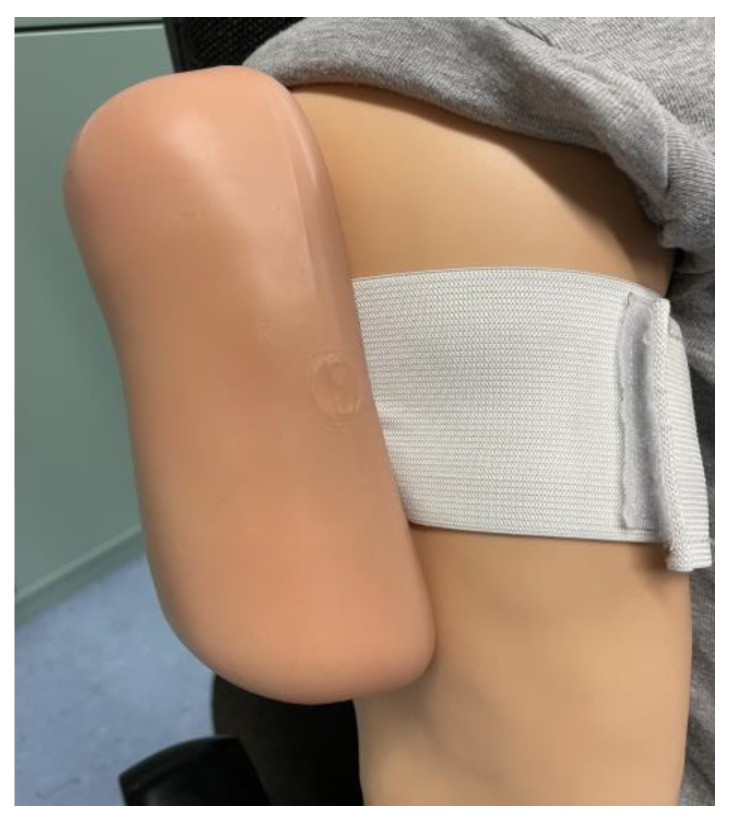
Low-fidelity Simulator.

In order to meet these—as well as future—challenges, vaccination administration training should be implemented into the curriculum of pharmaceutical education. Currently, pharmacists in at least 13 countries are required to complete additional training with respect to vaccination administration [14]. For instance, Australia and the USA have included teaching content related to immunization in the pharmacy curriculum [27,28]. The efficacy of a university course for the purposes of training pharmacy students in vaccine administration has been demonstrated and evaluated by many studies [29,30,31,32]. However, in Germany, additional training with defined content has been offered recently for the purposes of training pharmacists in a continuous education and training program [17]. The training covers the following areas: practical administration of the vaccination; obtaining patient information and consent; observing contraindications; as well as recognizing emergency situations and their measures [16]. The course consists of a theoretical and practical part, in which the injection is performed on a low-fidelity simulator (LFS)—i.e., a model arm or wearable pad with a tissue-like structure (Figure 1). In addition, participants have the option to perform an injection with a sodium chloride solution on a real person after obtaining consent, and the entire course is supervised by a trained doctor.

**Figure 2:**
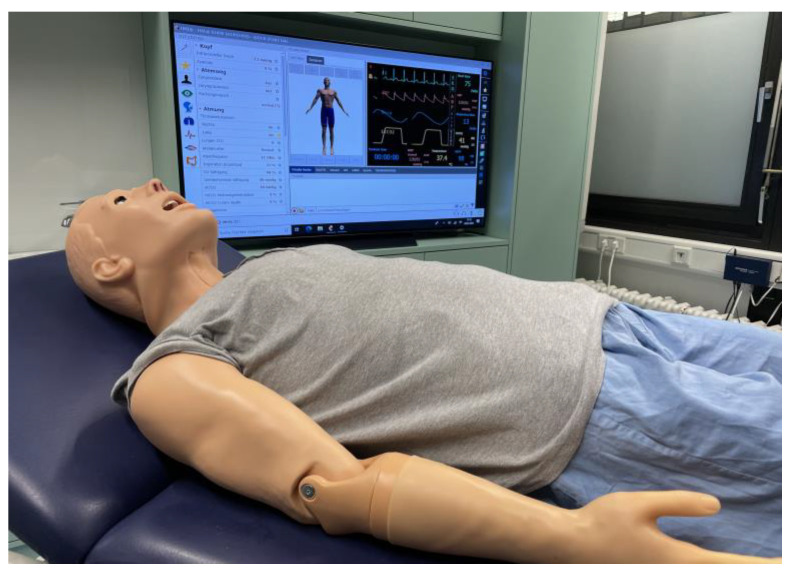
High-fidelity Simulator with Software showing vital functions.

Using simulation in the training of clinical skills can lead to improved knowledge, performance, and satisfaction among students and health-care professionals [33,34]. Further, high-fidelity simulation (HFS) is the most cutting-edge simulation technique currently available, whereby a mannequin is operated on via the use of a software that is utilized in order to simulate changes in physiological parameters [35] (Figure 2). HFS is intended to aid with developing practice and to demonstrate clinical skills (such as patient handling in critical situations or patient safety awareness in a risk-free and controlled environment [35]). This kind of simulation is already being utilized in medical and nursing education [36,37] and is also being utilized in pharmacy education in order to train various clinical skills, as well as to enhance student competence and knowledge [38,39,40]. The impact of this type of clinical skills training can be measured by the objective structured clinical examination (OSCE), which is a common and established method of assessing clinical skills [41,42,43]. 

Traditional pharmacy education regarding vaccines only provides didactic knowledge about vaccines and their administration. The authors were not aware of any practical vaccination training courses that were available to pharmacy students at German universities. Neither were there any documented evidence on the integration and impact of HFS for the purposes of training pharmacy students’ vaccination administration skills, nor in regard to the simulation of various emergency scenarios. Therefore, in the present study, the aim is to develop an innovative training course with an HFS approach that integrates emergency handling. In addition, whether the HFS leads to better performance in comparison to the standard training (which utilizes LFS) is investigated; further, this was achieved by using an analytical checklist for the purposes of evaluation. The primary endpoint is, therefore, to demonstrate the difference in performance; additionally, the secondary endpoint is to demonstrate the variations in the participants’ self-assessment and knowledge scores, if any. In this paper, we would like to present our work and have arranged this into methods, results and the following discussion.

## 2. Materials and Methods

### 2.1. Study Design and Participants

**Figure 3:**
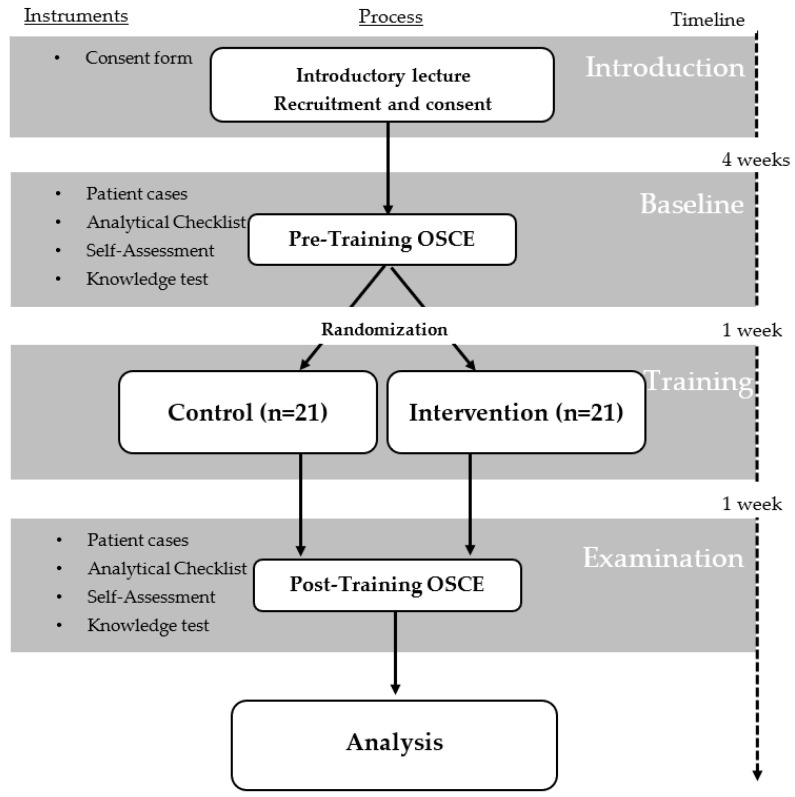
Flow Chart of the randomized controlled study. OSCE = objective structured clinical Examination.

A pre and post randomized controlled trial with pharmacy students was conducted in order to investigate the effect of the HFS training approach on vaccination administration skills. This was then followed by an OSCE evaluation. The investigation was carried out, in German, from November 2021 to December 2021 as part of the “Clinical Pharmacy” course in the winter semester of 2021/2022. All the data were collected in pseudonymous form and were anonymized in the following analysis. In addition, approval of this study was granted by the responsible ethics committee (Nr.: 2021–1689). In October 2021, 46 fourth-year pharmacy students were invited to participate in the study at the Heinrich-Heine-University in Duesseldorf. After completing the informed consent procedure, participants were randomized either into a control group or into an intervention group using RStudio (Version 1.4.1106) [44]. Participants were first sorted alphabetically and pseudonymized with “WS01” in increasing order. Then, the function “sample()” in the statistical software environment R was used to assign the participants to two equally sized groups A and B, where A was the control group and B the intervention group. Further, the overall study design is illustrated in Figure 3. 

### 2.2. Study Procedure

At the beginning, an introductory lecture on influenza vaccination was conducted in order to ensure the same level of theoretical knowledge among all students. The contents included background information on the influenza virus and influenza vaccination; worldwide community pharmacy-based vaccination practices; the current status and legal requirements for vaccination administration in Germany; and the possible role of a community pharmacist. On the same day, the participants were informed in detail about the study and the consent forms for participation were then distributed. After 4 weeks, the participants completed a pre-training OSCE, a multiple-choice test, and a self-assessment questionnaire. In the next week, the respective vaccination administration training was conducted, which included emergency scenarios that was initiated for both groups. The control group received the standard training by using injection pads. The intervention group was trained via undertaking the HFS approach [45]. Finally, one week later, the participants completed a post-training OSCE, a second multiple-choice test, and another self-assessment questionnaire.

### 2.3. Objective Structured Clinical Examination

Participants were assessed individually with respect to vaccination administration through pre- and post-training OSCEs. This was conducted with an intent to measure any differences in performance, if present. Five OSCE cases were prepared and reviewed by faculty members during focused group discussions. During the OSCEs, each participant was required to simulate a pharmacy-based vaccination administration process involving a standardized patient and was assessed by an observer who utilized an analytical checklist. A pharmacy-like environment was created for this purpose, where all necessary items were available. Participants were provided with individual time slots and received a brief description of the whole simulation process after registration. An OSCE lasted a maximum of 12 min. Faculty members and eighth-semester pharmacy students, who did not participate in the study, were trained and instructed to serve either as standardized patients or as observers. Standardized patients were replaced after each OSCE, while observers were replaced after every five OSCEs. After obtaining the participants’ consent, certain OSCEs were recorded for quality assurance purposes. Randomly selected videos were then evaluated independently by two faculty members and the checklist scores were adjusted, as necessary.

### 2.4. Training Sessions

The 2.5 h-long training sessions were conducted separately for each study group. Both groups received blended theoretical and practical training with respect to anamnesis; patient education and information; vaccination preparation and administration; potential emergency situations; and the necessary measures to deal with them. The control group was trained in vaccination administration skills via the standard approach using LFS, i.e., utilizing injection pads. The intervention group interacted with an HFS and injected the vaccine intramuscularly. In addition, participants could talk directly to the HFS via an integrated microphone. A faculty member controlled the simulator remotely and responded to the participants. Further, various emergency scenarios were simulated by changing vital parameters through an in-built software program.

### 2.5. Instruments

#### 2.5.1. High- and Low-Fidelity Simulator

For the purposes of training participants’ vaccination administration skills, two different kinds of simulators were employed. The control group practiced intramuscular injection using a wearable LFS injection pad with a tissue-like structure (Erler-Zimmer Impftrainer; Figure 1); it must be noted that, currently, standard training uses simulators of this kind. In order to simulate the insertion of a needle into tissue, the injection pad enables for proper placement around the arm, as well as simulating the actual depth of injection and withdrawal of the needle. The intervention group completed the vaccination administration training on an HFS (Gaumard HAL^®^ S1000; Figure 2). The simulator can be operated on by an in-built software and includes various controllable features. Important features include palpable pulse, heart and lung sounds, chest and abdominal movements, and an attached cuff for blood pressure measurement. A built-in microphone allows a person to speak directly through the mannequin to communicate with participants. An intramuscular injection can be performed on the upper arm. Changes of vital parameters, such as heart/respiratory rate or blood pressure, can be transferred either immediately or after specified time on the simulator.

#### 2.5.2. Cases for OSCEs

Five different patient cases with emergency scenarios were developed and reviewed by faculty members during focused discussion groups and faculty meetings. The emergency scenarios dealt with asthma exacerbation, hypoglycemic events, angina attacks, anaphylactic shock, and vasovagal syncope [45] following vaccine administration. All cases possessed the pattern of a patient coming to the pharmacy for a flu vaccination and an emergency arising after receiving the vaccination. In addition, a medication plan was also prepared for each case. In the case of an asthma attack and angina attack, there were also emergency medications, which the standardized patients carried with them. Specific checklists were prepared for each case, which differed in content only in Station 4 (which is related to emergency scenarios). Standardized Patients were faculty members or pharmacy students who were trained to imitate respective emergency situations with verbal and non-verbal cues. Particular attention was paid to acting breathing and case-specific symptoms. Pathological characteristics, according to the emergency situation, were given in a short case description (Supplemental Material S7).

#### 2.5.3. Analytical Checklist

In order to quantify the performance of the participants, they were assessed during the OSCEs by an observer using an analytical checklist. The analytical checklist was created by faculty members and thoroughly discussed during several meetings. However, it must be stated that the Federal Chamber of Pharmacists’ official guidelines for flu vaccination in community pharmacies were followed in order to ensure that all relevant points and steps were included [46]. The checklist was also reviewed by a medical specialist and consisted of four stations. Each station contained subcategories and subitems with different total scores. Station 1 was related to taking a patient’s medical history, with a total of 8 or 9 points, depending on the individual cases. This station also dealt with identifying the patient’s eligibility for receiving the vaccination. Station 2 comprised tasks related to providing patient education and the necessary information regarding the vaccine and vaccination process. Station 3 included the necessary hygienic measures for the preparation of vaccines, the preparation on a personal basis and of the premises, and vaccination administration tasks. Further, Stations 2 and 3 each contained 12 points. Finally, Station 4 possessed 7 achievable points, which included tasks related to recognizing emergency situations and, thus, taking the necessary course of action. If a respective subitem was fulfilled, 1 point was awarded and if not zero points were given. 

#### 2.5.4. Multiple-Choice Test

In order to determine the participants’ knowledge related to the influenza virus and the vaccination process, a multiple-choice test was developed consisting of five multiple-choice questions. There were different sets of questions for the pre- and post-training OSCEs.

#### 2.5.5. Self-Assessment Questionnaire

In order to ascertain the participants’ self-assessment regarding their competency and use of simulations in vaccine administration and pharmacy teaching, a pre- and post-training OSCE self-assessment questionnaire was developed. This consisted of nine questions with a 6-point Likert scale where 1 was full disagreement and 6 was full agreement. Questions 1 to 6 were related to personal ability regarding the vaccination process and questions 7 to 9 were related to the use of simulations in clinical skill-based pharmacy training sessions. The same survey was also completed in the pre- and post-training OSCEs.

### 2.6. Statistical Methods

In this study, the effects of the HFS training approach on vaccination administration skills compared to the standard training (with LFS methods) through OSCEs were analyzed. The results of these are given in percentages in order to ensure comparability between pre- and post-training OSCEs, as well as the intervention and control groups. Since proportions, i.e., percentages of achieved points in the OSCEs, were considered in the comparison of the performance of the intervention and the control group, non-parametric tests were used for these comparisons. More precisely, in order to measure the success between pre- and post-training OSCEs of the respective groups, a one-sided paired Wilcoxon signed-rank test with a significance level of alpha = 0.05 was performed. In order to determine the difference between the intervention group and the control group with respect to the pre- and post-training OSCEs, a one-sided Mann–Whitney test was performed with a significance level of alpha = 0.05. Hence, a *p*-value below 0.05 was considered significant. In addition, Microsoft Excel 2019 [47] was utilized for the purposes of data entry and OriginPro 2021 [48] for statistical analysis.

## 3. Results

### 3.1. Participant Characteristics

Forty-two students in their fourth year of pharmacy studies, after providing their informed consent, were available to participate in this study. Only one participant was excluded from the analysis of the self-assessment questionnaire due to missing information. Table 1 describes the participant characteristics for both the intervention and the control groups.

### 3.2. Analytical Checklist Score of OSCEs

**Figure 4:**
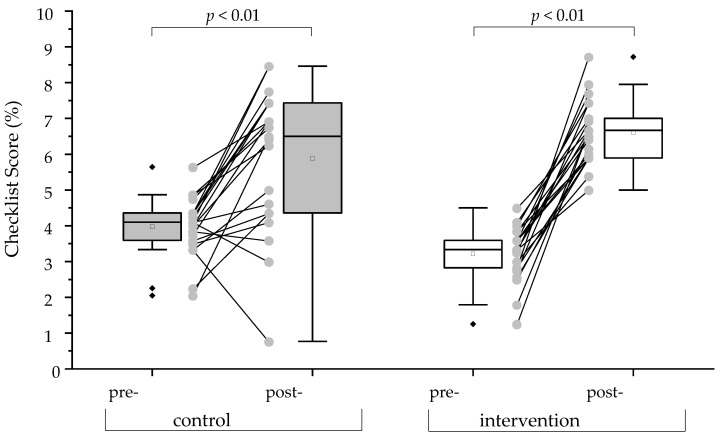
Box plots of analytical Checklist scores between pre- and post-Training OSCEs. The grey dots and lines show the difference in performance of each participant. The black diamonds (♦) indicate the outliers. A one-sided paired Wilcoxon signed-rank test with a significance level of alpha = 0.05 was used to compare OSCE scores between pre- and post-Training of the respective groups.

**Figure 5:**
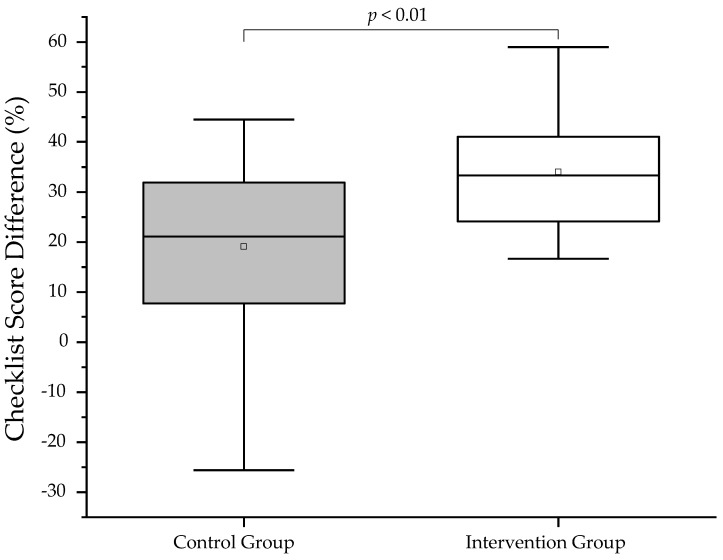
Box plots of analytical checklist score difference between pre- and post-Training OSCE. A one-sided Mann–Whitney test with a significance level of alpha = 0.05 was used to compare OSCE scores between groups.

**Figure 6:**
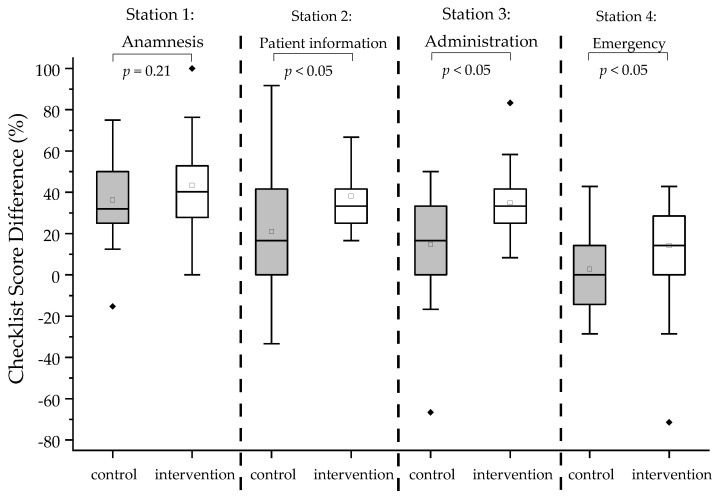
Box plots of analytical Checklist score differences for each station between pre- and post-training OSCE for respective groups. The black diamonds (♦) indicate the outliers. A one-sided Mann–Whitney test with a significance level of alpha = 0.05 was used to compare OSCE scores between groups.

The participants’ performance during the OSCEs was assessed and quantified using an analytical checklist. The analytical checklist scores reflect the participants’ ability to successfully conduct the vaccination process, i.e., from initiation to handling the untoward reactions. In total, 39 or 40 points could be achieved, depending on the individual case. The point-based scores were then converted to percentage points in order to enable comparisons between the groups and different OSCEs. For visualization of the data, box plots were generated. At baseline, the control group performed significantly better than the intervention group (*p* < 0.01; Table 2; Figure 4). Additionally, both groups demonstrated significant improvement in their overall performance from pre- to post-training OSCEs (intervention group: *p* < 0.01; control group: *p* < 0.01; Figure 4). However, the intervention group showed a significantly greater improvement in their analytical checklist scores when compared to the control group (*p* < 0.01; Figure 5). Accordingly, the intervention group showed significantly greater improvement in each station (*p* < 0.01 for station 2–4; Figure 6) except in Station 1, which is related to taking patient history, in comparison to the control group (*p* = 0.210 for station 1; Figure 6). It is interesting to note that every individual of the intervention group improved from pre- to post-training OSCEs (Figure 4). In Station 4, which is related to handling emergency situations, the intervention group demonstrated a significantly improved performance (*p*= 0.014) from pre- to post-training OSCEs when compared to the control group (*p*= 0.216).

### 3.3. Multiple-Choice Test 

The participants showed no significant (*p* = 0.471) increase in knowledge scores points in both groups from the pre- to post-training multiple-choice tests (see Table 3).

### 3.4. Self-Assessment Questionnaire 

**Figure 7:**
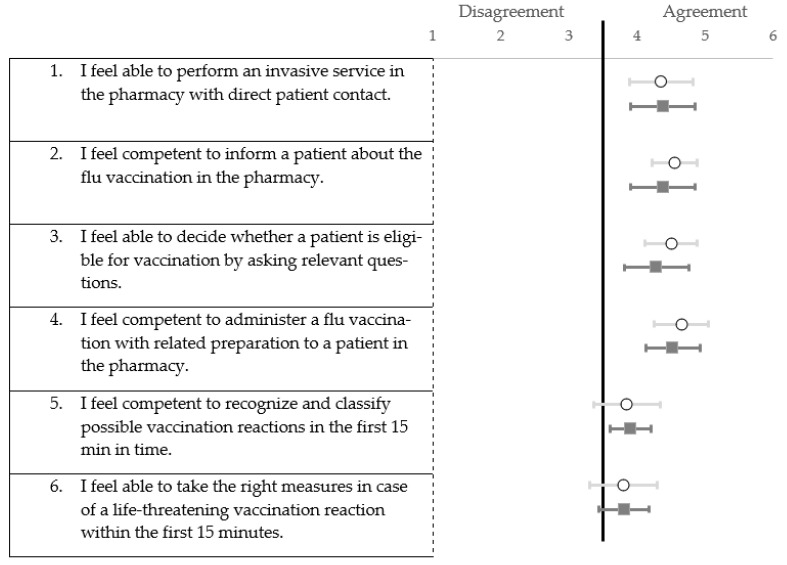
Forest plot of mean values with 95% confidence interval of self-assessment scores for question 1 to 6 in post-Training (6-point Likert scale). White dots (○) = intervention group (n = 20); grey square (**▪**) = control group (n = 21).

**Figure A1:**
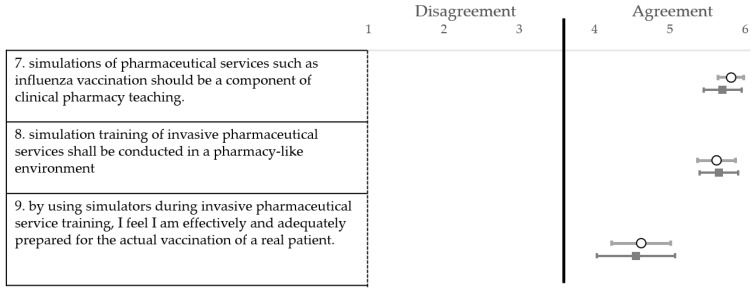
Forest Plot of mean values with 95% confidence interval of self-assessment scores for question 7 to 9 in post-Training (6-point Likert scale). White dots (○) = intervention group (n = 20); grey square (▪) = control group (n = 21).

Both groups demonstrated a similar increase in self-assessment scores when evaluated through a 6-point Likert scale. There were no significant differences between the intervention and control groups in regard to baseline (*p* = 0.505). This was also the case for the post-training self-assessment questionnaire scores (*p* = 0.568). Both groups reached significantly greater scores (intervention and control group: *p* < 0.01) from the pre- to post-training self-assessments. However, on question 5—which concerns the competency of recognizing and classifying possible vaccination reactions within the first 15 min after vacation—certain participants from the intervention group showed signs of disagreement (Figure 7). A few participants from both the intervention and control group disagreed with the statement regarding their self-efficacy for acting appropriately in an emergency situation during the first 15 min (Figure 7). Statistical computation of the questionnaire and results for question 7 to 9 are depicted in Appendix A (Table A1, Figure A1).

## 4. Discussion

In the present study, simulation-based training for pharmacy students in order to better educate them regarding vaccination administration was successfully implemented. In this study, positive students’ outcomes, as evidenced in the improved performance and overall self-assessment scores when compared to the control group who were trained in the standard teaching method, were demonstrated. Both groups significantly improved their performance from pre- to post-training. However, the participants of the intervention group with their HFS-based training were able to perform significantly better in terms of dealing with patient information, vaccination administration, and the handling of emergency situations. Importantly, the HFS training proved to be an effective teaching tool as every individual in the intervention group improved their performance; whereas in the standard training, some participants remained at the same level and some even decreased in ability.

High-fidelity simulation proved to be very effective for the purposes of vaccination training and resulted in significantly better outcomes in terms of participants’ performance. This may be attributed to the fact that HFS offers a patient-centered experience and can truly imitate the situation in a safe environment [49]. Other studies undertaking simulation-based vaccination training for pharmacy students showed similar results [50,51]. However, the comparability to our study is partially limited as they have not utilized a high-fidelity mannequin. For instance, Skoy et al. used two forms of simulators: an injection arm as a higher fidelity form of a simulator and an injection pad [51]. Similarly, Bushell et al. utilized roleplays, low-fidelity mannequins, standardized patients, and a mixed reality in order to create a realistic experience [50]. Both reported improved knowledge and confidence level among students. Furthermore, a key focus of this study was to train the participants in safe vaccination administration skills. Through the application of HFS in this study, there was an integration of different emergency cases—potentially arising after vaccination administration—that may or may not be directly related to vaccination, such as asthma exacerbation. As an aside, other studies on vaccination training have only focused on anaphylactic reactions [50,52,53]. Students in the HFS group particularly demonstrated significantly better performance in terms of recognizing and handling emergency situations. In addition, they also demonstrated a better performance in terms of the station dealing with patient information and vaccination administration. Abajas-Bustillo et al. found that communication skills could also be promoted by using HFS [54]. In addition, Tokunaga and colleagues demonstrated that students’ self-reports increased in understanding in monitoring vital signs and their measurements, including intramuscular injection [55]. In line with our findings, it could be shown that HFS training enhances competence with respect to specific clinical areas and skills. However, in contrast, Massoth et al. demonstrated that HFS did not lead to a significant improvement in performance, but the participants in this group were overconfident in their self-assessment [56].

We believe that through interaction with the HFS, clinical skills can be perceived and performed more efficiently. In our study, every single participant improved after training with the HFS, while certain participants who did not receive training with the HFS performed lower in their pre- to post-training OSCEs. Improved performances were, however, reported in several studies among nursing [57,58], medical [59], and pharmacy students [60,61,62]. This was specifically in regard to teaching clinical skills by the use of HFS. However, training should ultimately qualify and improve each participant. In a previous study, simulation performance was shown to correlate with clinical performance [63]. Thus, simulation performance was considered the best predictor of clinical performance [63]. Therefore, it seems appropriate that simulation-based learning should be included in the curriculum in order to teach clinical skills at an early stage. 

The fact that simulation-based teaching is generally accepted by students is also shown in the results of the self-assessment questionnaire. The questions seven to nine, regarding using simulations in pharmacy education, were all answered with a general agreement both before and after the respective trainings. It was surprising to note that despite the improved performances, certain students disagreed in their self-assessment regarding their ability in recognizing and handling emergency situations. This fact contrasts with the findings reported by Zamami. Y and colleagues. Their study demonstrated a higher confidence with respect to the participants in handling emergency patients after undertaking pharmaceutical life-saving skills training [64]. This lack of agreement in our study could be attributed to two factors. Firstly, the participants have had no patient interaction, nor exposure to clinical situations during their pharmacy studies in Germany; as such, they will still, therefore, possess a certain lack of self-assurance. Secondly, the training sessions were their first hands-on experience in which they had to make real time decisions in a close-to-real environment. Therefore, nervousness could be a possible reason for the low confidence level reported as well [62]. However, it must be stressed that these participants’ perception of their ability was at variance from their actual performance during the study. Their lack of confidence could be addressed by scheduling trainings at an early stage and assigning the students with a responsibility to vaccinate, such as what was demonstrated by Peter R. Caroll and colleagues when they developed a student-led vaccination clinic for the purposes of training medical, nursing, and pharmacy students [52].

We are aware that our study is subject to certain limitations. Firstly, the present study did not determine any significant increase in terms of knowledge among the participants. The questions asked in the pre-training OSCE, as well as in the post-training OSCE, were of a general nature and, therefore, are possibly solvable, independently of the training. Therefore, the results of the multiple-choice tests cannot be correlated with the results of performance. Secondly, the patients were played by pharmacists, technical assistants, or pharmacy students instead of professional actors. In order to avoid a possible bias, both instructive and demonstrative instructions were given in several sessions. These included, in addition to medical history and medication, acting out certain symptoms and signs for emergency situations. In addition, the actors were not the observers filling out the checklist. The reason for this was to keep the focus entirely on their role rather than on completing a checklist. Additionally, the actors were given at least 15 min between OSCEs in order to prepare for the next case. Thirdly, the observers were pharmacists or pharmacy students, and in order to avoid possible inter-observer scoring bias, the analytical checklist was discussed in several sessions and example sentences or actions were explained. Additionally, each participant was assessed by the same observer in the pre- and post-training OSCE. In order to maintain concentration, the observers changed after every five examinations. The students who assisted in conducting the OSCEs were involved in a pilot study and, therefore, participated as members of the study team. Fourthly, due to legal and ethical reasons, it was not possible to allow students to perform an injection on a real person, as is offered in the standard training. Additionally, the constant presence of a doctor for supervision is still difficult to facilitate. Therefore, we used standardized patients, who wore a vaccination pad around their upper arm during the OSCEs, in order to imitate a real vaccination process in a community pharmacy. In addition, a pharmacy-like environment was created in order to simulate the situation as realistically as possible. Fifthly, despite randomization, all participant with previous pharmaceutical work experience were in the control group. This could be a possible explanation, that the control group reached significant greater scores during pre-Training OSCEs compared to the intervention group. In addition, the difference in Station 2 in pre-OSCE between the two groups was very high. However, when asked about pharmaceutical work experience, participants were also asked to specify whether they had worked or were working as a pharmaceutical technical assistant, in vaccination centers or similar, or in other areas. None of the participants worked in vaccination centers or similar, excluding previous experience in this field. We performed a sensitivity analysis in this respect excluding the six participants with pharmaceutical work experience. All results remain the same except for Station 2 (patient information) where no statistically significant difference could be shown in terms of performance between the two groups. The results of the sensitivity analysis are given in the Supplementary Materials. Finally, the number of participants seems to be low, and the study was only conducted at one university. Therefore, a power analysis was performed using the resulting means, standard deviations, and sample sizes, which yielded a power of 92%. Therefore, even though number of participants seems to be low, a statistical power was reached that is larger than the usually targeted power of 80 or 90%. However, further studies with a larger number of participants as well as the inclusion of more universities are recommended. Moreover, the training courses employing the high-fidelity simulator can be very expensive [37], especially for low-income countries where there is a high need for pharmacist-led vaccination administration. Collaborations at the university or even at the national level can address this issue, as the simulator can be transported. This could also increase vaccination rates in poorer or rural communities.

## 5. Conclusions

Pharmacists are becoming increasingly involved in vaccination in Germany as well as in many other countries. In order to ensure safe vaccinations being conducted by pharmacists, training with HFS proved to be superior to standard training in this study. Particularly, it could be shown that emergency situations can be addressed in a very effective manner. Furthermore, the introduction of such a course into the pharmacy curriculum should be considered in order to prepare students for future challenges. 

## Figures and Tables

**Table 1:** Participant Characteristics.

	Control Group (n = 21)	Intervention Group (n = 21)	*p*-Values
**Age**			
Mean (±SD) Median Range	25 (±2.67) 24 22–32	24.38 (±2.35) 23 22–31	0.337
**Gender**			
Female, n (%) Male, n (%)	17 (81) 4 (19)	18 (86) 3 (14)	1
**Previous or current experience (e.g., pharmaceutical technician, vaccination centre)**			
Yes (%) No (%)	6 (29) 15 (71)	0 (0) 21 (100)	0.021

**Table 2:** Achieved scores by intervention and control group in each station of analytical checklist during pre- and post-Training OSCEs.

Group	Pre-Training OSCE-Score	Post-Training OSCE-Score	Score Difference
	Mean (SD) %	Mean (SD) %	Mean (SD) %
**Station 1**			
Intervention	26.06 (14.81)	69.38 (15.72)	43.34 (23.18)
Control	27.65 (18.53)	63.89 (18.92)	36.24 (21.54)
**Station 2**			
Intervention	7.54 (9.83)	45.64 (17.00)	38.10 (16.79)
Control	22.22 (13.26)	43.25 (28.34)	21.03 (32.13)
**Station 3**			
Intervention	49.60 (15.47)	84.52 (11.87)	34.92 (17.60)
Control	53.18 (17.38)	67.86 (22.10)	14.68 (26.10)
**Station 4**			
Intervention	51.70 (19.42)	65.99 (17.77)	14.29 (27.85)
Control	61.22 (26.40)	63.95 (27.71)	2.72 (19.50)
**Total**			
Intervention	32.10 (7.70)	66.12 (8.89)	34.03 (11.66)
Control	39.73 (8.21)	58.79 (19.84)	19.06 (18.20)

Depending on case, different maximum scores were achievable. (OSCE = objective structured clinical examination, SD= standard deviation).

**Table 3:** Achieved scores by intervention and control group in pre- and post- training multiple-choice-test.

Group	Pre-Training	Post-Training
	Mean (%)	Mean (%)
**Intervention**	3.00 (60)	3.43 (68.57)
**Control**	2.7 (55.24)	2.86 (57.14)

n = 21 each for intervention and control group.

## Data Availability

The dataset presented in this study is available from the corresponding author on reasonable request.

## Supplementary Materials

The following supporting information can be downloaded at: https://www.mdpi.com/article/10.3390/vaccines11020324/s1, Supplemental Material S1: OSCE-Checklist Station 1–3, Supplemental Material S2: OSCE-Checklist Station 4 case 1, Supplemental Material S3: OSCE-Checklist Station 4 case 2, Supplemental Material S4: OSCE-Checklist Station 4 case 3, Supplemental Material 5S: OSCE-Checklist Station 4 case 4, Supplemental Material S6: OSCE-Checklist Station 4 case 5, Supplemental Material S7: short case descriptions and Medication Plan Supplemental Material S8: Multiple Choice Tests; Supplemental Material S9: sensitivity analysis.

## Appendix A

**Table A1:** Achieved scores by intervention and control group in pre- and post-training self-Assessment.

Group	Pre-Training	Post-Training	*p*-Value (Intragroup)
	Mean (CI)	Mean (CI)	
**Intervention**	32.45 3.10	41.60 2.77	*p* < 0.01
**Control**	33.92 2.96	41.33 2.10	*p* < 0.01
***p*** **-Value** **(intergroup)**	*p* = 0.505	*p* = 0.568	

n = 20 for intervention group and n = 21 for control group; CI = Confidence interval (α = 0.05).
