# Supplementary material for: Development and Assessment of Innovative High-Fidelity Simulation Vaccination Course Integrating Emergency Cases for Pharmacy Undergraduates—A Randomized Controlled Study
Source: Vaccines (Basel). 2023 Jan 31;11(2):324. doi: 10.3390/vaccines11020324 (PMC9967866; doi:10.3390/vaccines11020324)
Supplement: Supplementary file 1 [file vaccines-11-00324-s001.zip › vaccines-2160883-supplementary.pdf]

Supplemental Material S1: OCSE-Checklist station 1 -3

Station 1: Anamnesis (9P.)			fulfilled	Not fulfilled
1.1	*Medical history: The examinee asks about acute and chronic diseases and excludes contraindications*	acute diseases/infection		
		chronic diseases		
		pregnancy (only for female patients)		
		planned surgery in the next 3 days		
		fever (>38.5°C)		
1.2	*Medication history: the examinee asks about all medications taken.*	previous/current medication (Rx, OTC, NEM)		
		taking blood thinners		
1.3	*Allergy: examinee asks about previous and current allergies*	known allergies		
		allergic to vaccines/components (previous vaccination reaction)		

Station 2: Patient Information/Counseling (12P.)				
2.1	*Influenza Infection & Treatment Options: The examinee educates the patient generally and about possible risks of influenza infection*	Symptoms of influenza infection		
		Routes of infection		
		Risk group(s)		
		Season		
2.2	*Vaccination: The examinee educates the patient about the benefits and administration of vaccination.*	Benefits		
		UAW/Complications		
		Vaccination administration process		
		complete vaccination protection		
2.3	*Other: The examinee....*	clarifies open questions		
		hands out information leaflet		
		has consent and privacy statement signed		
		points out OTC medications		

Station 3: Vaccination administration(13P.)				
3.1	*Preparation: The examinee makes all preparations for the administration of the vaccination.*	Area preparation (e.g. disinfection)/provision of materials		
		Preparation of own person (e.g. washing hands, gloves)		
		Vaccination (tempering, shaking, testing the syringe)		
		Patient preparation (position, selection of the arm)		
3.2	*Execution: The examinee administers the vaccination in the correct manner.*	Disinfection of the injection site		
		Technique (3-finger rule, 90° injection)		
		rapid and complete injection		
		Formation of a skin fold and care of the injection site (plaster)		
		Disposal		
3.3	*Instructions & documentation: The examinee gives the patient the correct instructions and performs the relevant documentation*	15 min waiting/observation time		
		Vaccination certificate (for the patient)		
		Vaccination documentation (for the pharmacy)		

Supplemental Material S2: OSCE-Checklist station 4 case 1

Station 4: Scenario - Anaphylactic reaction (7P.)				
4.1	*Subjective: The examinee first addresses the patient's complaints*	Checks responsiveness/responds to the patient		
		asks about symptoms/complaints		
4.2	*Objective: The examinee collects relevant parameters*	recognizes itching/urticaria in the patient		
		takes blood pressure/heart rate		
4.3	*Assessment: the examinee correctly classifies the emergency situation*	detects allergic reaction (low grade)		
4.4	*Plan: The examinee initiates the necessary measures*	makes emergency call		
		First aid measures (change position, provide fresh air, keep calm, attend to patient)		

Supplemental Material S3: OSCE-Checklist station 4 case 2

Station 4: Scenario - vasovagal syncope (7P.)				
4.1	*Subjective: The examinee first addresses the patient's complaints*	Checks responsiveness/responds to the patient		
		Asks about symptoms/complaints		
4.2	*Objective: The examinee collects relevant parameters*	obtains blood pressure/heart rate		
4.3	*Assessment: the examinee correctly classifies the emergency situation*	recognizes vasovagal syncope		
4.4	*Plan: The examinee initiates the necessary measures*	makes emergency call		
		first aid measures (change of position, fresh air supply, keep calm, care)		
		puts the patient's legs up		

Supplemental Material S4: OSCE-Checklist station 4 case 3

**Station 4: Scenario - Angina pectoris (7P.)**				
4.1	*Subjective: The examinee first addresses the patient's complaints*	Checks responsiveness/responds to the patient		
		Asks about symptoms/complaints		
4.2	*Objective: The examinee collects relevant parameters*	Takes blood pressure/heart rate		
4.3	*Assessment: the examinee correctly classifies the emergency situation*	recognizes angina pectoris attack		
4.4	*Plan: The examinee initiates the necessary measures*	makes emergency call		
		first aid measures (change of position, fresh air supply, keep calm, care)		
		advises use of nitro spray		

Supplemental Material S5: OSCE-Checklist station 4 case 4

Station 4: Scenario - Asthma attack (7P.)				
4.1	*Subjective: The examinee first addresses the patient's complaints*	Checks responsiveness/responds to the patient		
		Asks about symptoms/complaints		
4.2	*Objective: The examinee collects relevant parameters*	takes blood pressure/heart rate		
4.3	*Assessment: the examinee correctly classifies the emergency situation*	recognizes asthma attack		
4.4	*Plan: The examinee initiates the necessary measures*	makes emergency call		
		first aid measures (change of position, fresh air supply, keep calm, care)		
		advises use of asthma spray		

Supplemental Material S6: OSCE-Checklist station 4 case 5

Station 4: Scenario - Hypoglycemia (7P.)				
4.1	*Subjective: The examinee first addresses the patient's complaints*	checks responsiveness/responds to the patient		
		Asks for symptoms/discomforts (feeling warm, headache)		
4.2	*Objective: The examinee collects relevant parameters*	takes blood pressure/heart rate		
4.3	*Assessment: the examinee correctly classifies the emergency situation*	recognizes hypoglycemia		
4.4	*Plan: The examinee initiates the necessary measures*	makes emergency call		
		First aid measures (change position, provide fresh air, keep calm, attend to patient)		
		provides glucose		

Supplemental Material S7: short case description

Scenario 1: <u>Anaphylactic reaction</u>

Age	30 years
Profession	Teacher
Diseases	Allergic asthma
Medication	Salbutamol, Cetirizine
Contraindication	None
Additional information	Smokes sometimes, BMI: 23 kg/m²
Questions/Statements	How tolerable is the vaccination?
adverse reaction	Itching on the body, headache, feeling of heat, sweating<br>Question: What is wrong with me? What happened to me?<br>Respiratory rate: 25/min; Heart rate: 120/min; Blood pressure: 90/60 mmHG

Medikationsplan

Seite __ von __

für: geb. am:

ausgedruckt von:

(Stempel) ausgedruckt am:

Wirkstoff	Handelsname	Stärke	Form	Mo	Mi	Ab	zN	Einheit	Hinweise	Grund
Salbutamol	Salbuhexal	100µg	SUS						if needed	
Cetirizin	Cetirizin ADGC	10mg	TAB			1				

Scenario 2: <u>vasovagal Syncope</u>

Age	65 years
Profession	Cab driver
Diseases	Hypertension, diabetes, mild heart failure
Medication	Bisoprolol, metformin, ramipril, simvastatin
Contraindication	None
Additional information	Smokes more often, BMI: 26 kg/m²
Questions/Statements	I am afraid of injections.
adverse reaction	Dizziness, blackness in front of eyes, eyelids closing, speaks indistinctly, almost falls off the chair/ about to fall over<br>Question: What is wrong with me? What happened to me?<br>Respiratory rate: 30/min; Heart rate: 45/min; Blood pressure: 100/70 mmHG

Medikationsplan

Seite __ von __

für: geb. am:

ausgedruckt von:

(Stempel) ausgedruckt am:

Wirkstoff	Handelsname	Stärke	Form	Mo	Mi	Ab	zN	Einheit	Hinweise	Grund
Bisoprolol	BisoLich	5mg	TAB	1						
Ramipril	RamiLich	5mg	TAB	1						
Simvastatin	SimvaHexal	40mg	TAB			1				
Metformin	Metformin Heumann	850mg		1		1				

Scenario 3: Angina pectoris

Age	45 years
Profession	Factory worker
Diseases	CHD, hypercholesterolemia, hypertension,
Medication	ramipril, bisoprolol, simvastatin, nitrospray
Contraindication	None
Additional information	BMI: 28 kg/m²
Questions/Statements	Vaccination recommended by the doctor
adverse reaction	Stinging/pressure in chest (grabs chest), shortness of breath, gagging feeling in throat, nausea.<br>Question: What is wrong with me? What has happened to me?<br>Breathing rate: 30/min; Heart rate: 90/min; Blood pressure: 150/95 mmHG

Medikationsplan

Seite __ von __

AKTIONSPLAN ZUR VERBESSERUNG DER ARZNEIMITTEL THERAPIESICHERHEIT IN DEUTSCHLAND

für: ______ geb. am: ______

ausgedruckt von:

(Stempel) ausgedruckt am: ______

Wirkstoff	Handelsname	Stärke	Form	Mo	Mi	Ab	zN	Einheit	Hinweise	Grund
Bisoprolol	BisoLich	5mg	TAB	1						
Ramipril	RamiLich	5mg	TAB	1						
Simvastatin	SimvaHexal	40mg	TAB			1				
Glycerolnitrat	Nitrolingual akut	0,4mg	SPR						in emergency	

The highlighted medication was the emergency medication the standardized patient carried with them.

Scenario 4: Asthma attack

Age	25 years
Profession	Student
Diseases	Asthma
Medication	Salbutamol, fluticasone spray
Contraindication	None
Additional information	BMI: 21 kg/m²
Questions/Statements	Why should you get a flu shot?
adverse reaction	Difficulty exhaling (sounds when breathing out), dry cough, tightness in the chest.<br>Question: What is wrong with me? What has happened to me?<br>Respiratory rate: 23/min<br>Heart rate: 100/min; Blood pressure: 95/65 mmHG

Medikationsplan

Seite __ von __

AKTIONSPLAN ZUR VERBESSERUNG DER ARZNEIMITTEL THERAPIESICHERHEIT IN DEUTSCHLAND

für: ______ geb. am: ______

ausgedruckt von:

(Stempel) ausgedruckt am: ______

Wirkstoff	Handelsname	Stärke	Form	Mo	Mi	Ab	zN	Einheit	Hinweise	Grund
Salbutamol	Salbuhexal	100µg	SUS						if needed	
Beclometason/ Formoterol	Foster 100/6	10mg	INH	1		1				

The highlighted medication was the emergency medication the standardized patient carried with them.

Scenario 5: Hypoglycemia

Age	80 years
Profession	Retired
Diseases	Hypertension, osteoarthritis, diabetes (type II)
Medication	Insulin (short, long), bisoprolol, ibuprofen, zopiclone
Contraindication	None
Additional information	BMI: 28 kg/m²
Questions/Statements	Has not been able to eat much today due to excitement.
adverse reaction	Confused, trembling, visual disturbance, decreased responsiveness, short unclear answers.<br>Question: what's wrong with me? What happened to me?<br>Respiratory rate: 10/min; Heart rate: 80/min; Blood pressure: 90/60 mmHG

Medikationsplan

Seite __ von __

AKTIONSPLAN ZUR VERBESSERUNG DER ARZNEIMITTEL THERAPIESICHERHEIT IN DEUTSCHLAND

für: ______ geb. am: ______

ausgedruckt von:

(Stempel)

ausgedruckt am: ______

Wirkstoff	Handelsname	Stärke	Form	Mo	Mi	Ab	zN	Einheit	Hinweise	Grund
Bisoprolol	BisoLich	5mg	TAB	1						
Ibuprofen	Ibuflam	600mg	TAB	1	1	1				
Zopiclon	Zopiclon AbZ	7,5mg	TAB			1				
Insulin (kurz)	Actrapid	40 IE	INJ						according to plan	
Insulin (lang)	Lantus	500 IE	INJ						according to plan	

Supplemental Material S8: knowledge quiz

Pre-training

1.) Which statement about the vaccination process is true?
- (A) The vaccine must be administered quickly after taking it out of the refrigerator, because the vaccine becomes ineffective due to stability problems.
- (B) The needle should be injected at a 45° angle to avoid damaging lower skin layers.
- (C) The injection should proceed rapidly.
- (D) After injection, pressure should not be applied to the injection site, as this may inhibit distribution of the vaccine.

2.) Which statement about possible vaccination reactions is true?
- (A) Systemic symptoms such as fever or headache can be treated with traditional OTC medications.
- (B) Side effects or vaccine adverse reactions do not occur with administration of a dead-vaccine because no viable pathogens are contained.
- (C) Typical symptoms of syncope include increased blood pressure and rapid pulse.
- (D) An allergic reaction may be manifested only by redness at the injection site.

3.) Which statement about vaccination and vaccines is correct?
- (A) Vaccination against influenza provides protection for at least 3 years.
- (B) The maximum protective effect of influenza vaccination is reached after only 5 days.
- (C) Influenza vaccines available on the market are exclusively administered intramuscularly.
- (D) Vaccines are usually added adjuvants, so-called effect enhancers.

4.) Which statement about rules and regulations of vaccinations is true?
- (A) The documentation for the administration of an influenza vaccination must be archived in the pharmacy for 3 years.
- (B) The Robert Koch Institute is responsible for approving vaccines.
- (C) Physician specialists may administer a vaccination regardless of the limits of their professional practice as a specialist.
- (D) The permanent vaccination commission is composed by new experts at each scheduled meeting.

5.) Which statement about influenza/illness and influenza vaccination in the pharmacy is true?
- (A) Neuraminidase inhibitors are also useful for prevention against influenza infection.
- (B) Antigenic drift requires multiple infection of the host cell.
- (C) Influenza vaccine is produced only via cell culture.
- (D) Taking blood thinners (e.g., Marcumar) is not an contraindication to administering vaccination at the pharmacy.

Post-training

1.) Which statement about the vaccination process is true?
- (A) The left arm should preferably be used to administer a vaccination.
- (B) Age is not a factor in the selection of influenza vaccine.
- (C) The injection cylinder should not be shaken before use, otherwise the vaccine may denature.
- (D) Before vaccination, the surfaces which are used should be disinfected.

2.) Which statement about possible vaccination reactions is true?
- (A) Headache and/or myalgia are very common side effects in people over 60 years of age.
- (B) Anaphylactic reactions are classified into five grades.
- (C) In case of syncope, the patient should be kept in an upright position to prevent a possible fall.
- (D) After 15 minutes of waiting, there is no longer a risk of vaccine reactions occurring in the patient.

3.) Which statement about vaccination and vaccines is correct?
- (A) Subunit or split vaccines are types of Live vaccines.
- (B) Multiple doses of vaccine can be obtained from one chicken egg.
- (C) Vaccines against two or more diseases should not be applied at the same time.
- (D) The purpose of a vaccination is to produce immunoglobulins against pathogens in the body.

4.) Which statement about rules and regulations of vaccinations is true?
- (A) There must be a 2-week interval between an influenza vaccination and Covid-19 vaccination.
- (B) Reports of ADRs after receiving a vaccination are received by the Robert Koch Institute.
- (C) The Infection Control Act should establish uniform regulations for the prevention of communicable diseases.
- (D) The European target for vaccination coverage among at-risk groups is set at 65%.

5.) Which statement about influenza/illness and influenza vaccination in the pharmacy is true?
- (A) For anxious patients, it is best to give the vaccination standing up.
- (B) New influenza subtypes may emerge due to antigenic shift.
- (C) Only inactivated influenza vaccines are available on the German market.
- (D) The membrane protein neuraminidase initiates endocytosis of the virus into the host cell.

Supplemental Material S9: sensitivity analysis

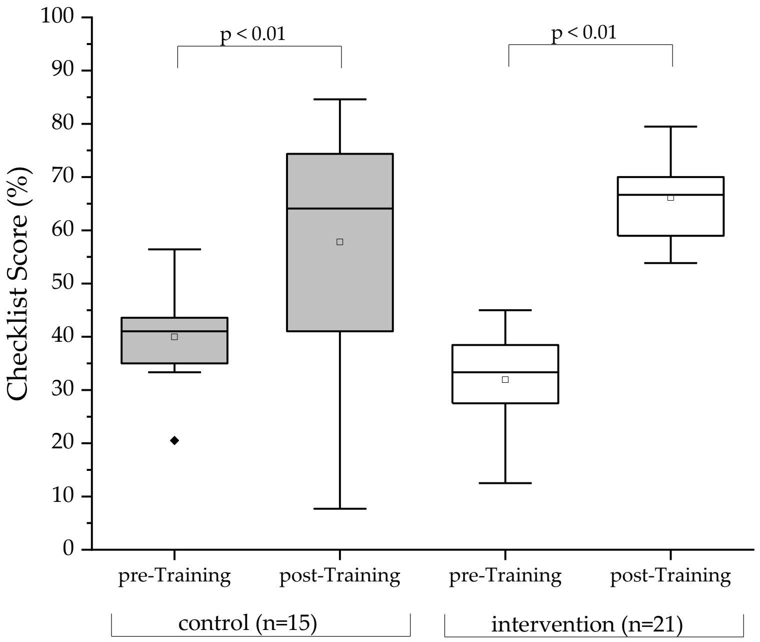

Figure S1. Box-Plots of analytical Checklist scores between pre-Training and post-Training OSCEs. The black diamonds (♦) indicates the outliers. A one-sided paired Wilcoxon signed-rank test with a significance level of alpha=0.05 was used.

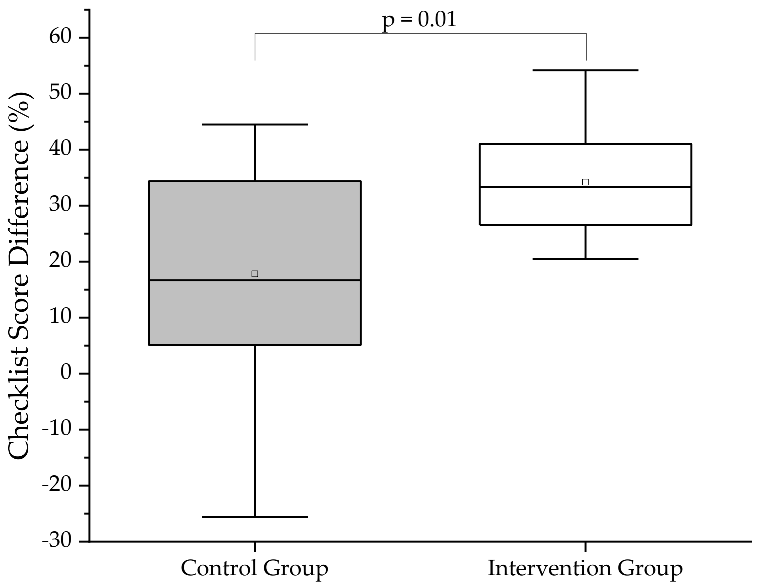

Figure S2. Box-Plots of analytical checklist score difference between pre-training and post-Training OSCE. A one-sided Mann-Whitney test with a significance level of alpha=0.05 was used.

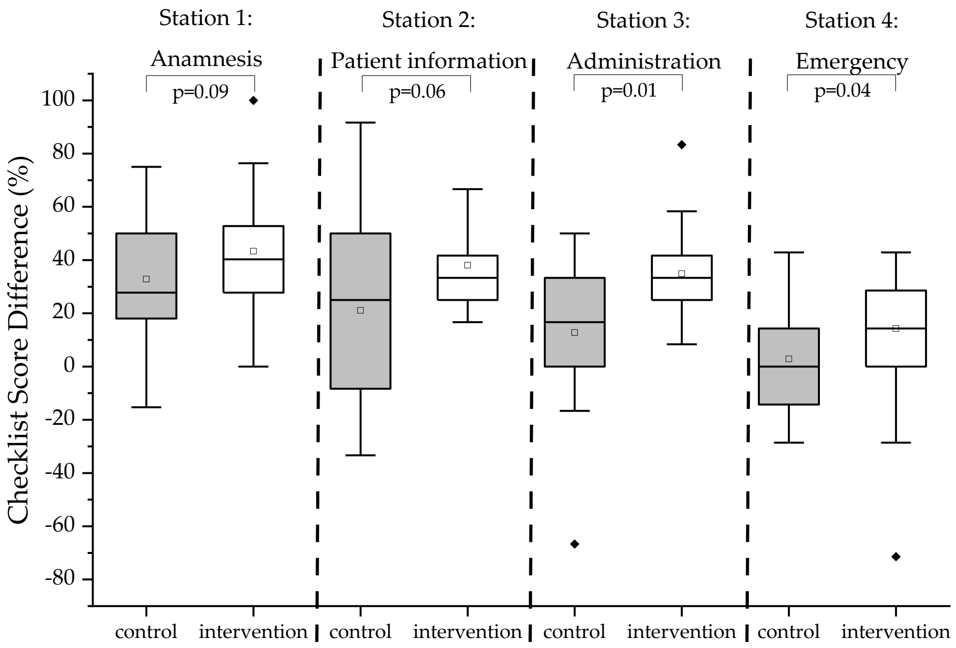

Figure S3. Box-Plots of analytical Checklist score differences for each station between pre-training and post-training OSCE for respective groups. the black diamonds (♦) indicate the outliers. A one-sided Mann-Whitney test with a significance level of alpha=0.05 was used.
